# Caffeine and preterm infants: multiorgan effects and therapeutic creep: scope to optimise dose and timing

**DOI:** 10.1038/s41390-025-04066-1

**Published:** 2025-06-20

**Authors:** Michael O’Shea, Luke Butler, Sam Holohan, Kate Healy, Rebecca O’Farrell, Amreena Shamit, Ruth Cusack, Mai Elhadi, Sinead Lynch, Megan Gilcrest, Jana Semberova, Aoife Branagan, Mary Isabel O’Dea, Peter Duddy, Namasivayam Ambalavanan, Karel Allegaert, Cynthia F. Bearer, Judith Meehan, Eleanor J. Molloy

**Affiliations:** 1 Paediatrics, School of Medicine, Trinity College, The University of Dublin, Dublin, Ireland; 2 School of Medicine, University College Dublin, Dublin, Ireland; 3 Neonatology, Coombe Hospital, Dublin, Ireland; 4 Trinity Research in Childhood Centre (TRICC), Trinity College Dublin, Dublin, Ireland; 5 Pharmacy, Coombe Hospital, Dublin, Ireland; 6 University of Alabama at Birmingham, Birmingham, AL USA; 7 Department of Development and Regeneration, KU Leuven, Leuven, Belgium; 8 Department of Pharmaceutical and Pharmacological Sciences, KU Leuven, Leuven, Belgium; 9 Department of Clinical Pharmacy, Erasmus Medical Center, Rotterdam, the Netherlands; 10 UH Rainbow Babies & Children’s Hospital, Cleveland, OH USA; 11 Case Western Reserve University School of Medicine, Cleveland, OH USA; 12 Paediatric Neurodisability, Children’s Hospital Ireland (CHI) at Tallaght, Tallaght University Hospital, Dublin, Ireland; 13 Neonatology, CHI at Crumlin, Dublin, Ireland

## Abstract

**Abstract:**

Caffeine is a methylxanthine used for nearly 50 years in the treatment of apnoea of prematurity (AOP). Caffeine citrate is effective in the treatment of AOP using standard dosing (loading dose 20 mg/kg, maintenance 5–10 mg/kg/day) and is associated with long-term neurological benefits and other improved organ outcomes as well as immunomodulatory effects. Therapeutic creep has been noted in the use of caffeine in preterm infants differing from the criteria in randomised controlled trials. A Cochrane review showed insufficient evidence to support prophylactic use of caffeine citrate in preterm neonates to prevent AOP, although it is still recommended in many national and local guidelines. Concerns about adverse reactions exist with high-dose caffeine regimens with one high-dose trial reporting statistically significant increases in abnormal neurological outcomes compared with standard doses (80 mg/kg compared to 20 mg/kg). International clinical guidelines vary from clinical trials regarding timing, dose, and duration of caffeine therapy. Further clinical research could help to understand optimal doses for different indications, such as peri-extubation, early postnatal use while ventilated, multiorgan and psychoactive effects, and long-term neurodevelopmental outcomes. This review describes the mechanism and multiorgan effects of caffeine highlighting areas of therapeutic creep and uncertainty requiring further research, such as comparative effectiveness trials.

**Impact:**

- Caffeine citrate is indicated for the management of apnoea of prematurity.
- Therapeutic creep is evident in international guidelines for the use of caffeine citrate in preterm infants.
- Caffeine has multiorgan effects involving renal, respiratory, and inflammatory responses, which, by optimising dosing and timing, may improve outcomes.
- Optimising indications, dose, and timing of caffeine citrate in preterm infants in further large-scale trials is warranted and may have other multiorgan benefits.

## Introduction

Caffeine in preterm infants has been used for nearly 50 years in the treatment of apnoea of prematurity (AOP).^1^ An apnoeic spell is usually defined as a cessation of breathing for 20 s or longer or a shorter pause accompanied by bradycardia (<100 beats per minute), cyanosis, or pallor. AOP occurs due to immaturity of the respiratory control mechanism and is triggered by events such as hypoxaemia and infection. Decreased gestational age and low birth weight are the most significant risk factors for developing apnoea.^1^ Initially characterised in the late 1960s, AOP is most common in neonates aged <28 weeks and weighing <1000 g, and occurs in at least 85% of infants born before 34 weeks of gestation.^2^

Prior to the advent of methylxanthine therapy, early cases of AOP were treated with mechanical ventilation and supplemental oxygen with frequent recurrence following discontinuation of therapy.^3^ Aminophylline was the first methylxanthine to be used for apnoea in the early 1970s.^3^ Caffeine and theophylline were subsequently introduced, supported by randomised controlled trials (RCTs) and Cochrane reviews.^3,4^ Caffeine citrate became commercially available following Food and Drug Administration approval in 1999 and in the EU in 2009 and has become the treatment of choice.^3^ Therapeutic creep in caffeine use has been described from clinical trial inclusion criteria to early caffeine use and the optimal doses, indications, efficacy, multiorgan effects, and adverse outcomes related to caffeine use in preterm infants. Therapeutic creep is the slow extension of treatments despite a lack of consensus or established guidelines to treat milder cases of a condition. This review aims to assess the evidence for the organ-specific function of caffeine in neonates and therapeutic creep.

## Systemic multiorgan effects of caffeine

Caffeine acts both centrally and peripherally, as a central nervous system stimulant and to enhance respiratory effort.^5–7^ The mechanism of action of caffeine in apnoea treatment is not fully understood, but several hypotheses have been suggested. Centrally, caffeine stimulates the medullary respiratory centre and increases sensitivity to hypercapnia.^5,7–10^ Physiologically, caffeine increases skeletal muscle tone, improves minute ventilation, increases oxygen consumption and metabolic rate, enhances diaphragmatic contractility and reduces fatigue.

Pharmacologically, these effects have been attributed to non-selective and selective adenosine antagonism at the adenosine A1 and A2a receptors to stimulate the medullary respiratory centre. Adenosine is important to maintain adequate adenosine triphosphate in the brain and increases markedly in hypoxia–ischaemia (HI) and endotoxemia modulating anti-inflammatory responses, vasodilation and endothelial leakage.^11^ Adenosine and other purine metabolites can be both protective and damaging depending on the activation of different adenosine receptors (e.g., A1, A2a, A2b, and A3) in different conditions.^12–14^ In addition, in infants with neonatal encephalopathy systemic adenosine correlated with severity and seizures.^15^ Therefore, modulating adenosine levels may have therapeutic benefits as well as acting as a biomarker.

Other methylxanthines such as theophylline have been used in the past for apnoea treatment, but caffeine is more effective and safer.^5,16^ A 2010 Cochrane Neonatal review comparing caffeine and theophylline concluded that both agents had similar efficacy, but theophylline demonstrated a higher rate of side effects, such as tachycardia and feeding intolerance.^16^ Caffeine also has superior enteral absorption, longer half-life and a wider therapeutic window when compared to theophylline.^16^

Caffeine has slower urinary excretion of un-metabolised drugs at earlier gestational ages and the serum half-life ranges from 40 to 230 h, which is >17-fold greater than adults. The half-life decreases to ∼2–4 h by 6–8 months of age.^17^ In view of the long half-life and the fact that caffeine may persist in the plasma for days after the completion of therapy, delayed apnoea can be seen up to a week following cessation. In neonates and young infants, caffeine is almost exclusively eliminated by renal clearance, which is affected by gestational age, parenteral nutrition, renal immaturity and dysfunction and co-morbidities.^17^

There is a large variation in caffeine metabolism between individuals and caffeine-metabolising enzymes, partly explained by genetic polymorphisms. Caffeine is rapidly disseminated through the body and crosses the blood–brain barrier. It is metabolised in the liver by cytochrome P-450 especially CYP1A2 and higher caffeine levels are found in those with a variant of the gene encoding this enzyme resulting in a slower caffeine metabolism.^18^

## Multiorgan effects of caffeine (Fig. 1)

### Caffeine and neuroprotection

Caffeine is a potent free radical scavenger and adenosine receptor antagonist that has been associated with reduced rates of brain damage in preterm infants. Neuroprotection and improved neurodevelopmental outcomes with decreased intraventricular haemorrhage (IVH) were shown in the “Caffeine for Apnoea of Prematurity” (CAP) trial with significantly less motor impairment in the caffeine-treated group versus the placebo at 11–12 years of age.^19^ Improved visuomotor, visuoperceptual and visuospatial abilities were also found at 11 years following neonatal caffeine treatment in the CAP trial.^20^ In addition, in a subgroup of 70 infants in the CAP trial, magnetic resonance imaging (MRI) at term corrected age revealed that caffeine-treated infants had improved white matter microstructural development compared to the placebo group.^21^ Eleven-to-12-year-old children in the CAP trial (*n* = 821) were assessed for health-related quality of life with no significant differences in most aspects although children with neuromotor impairment had lower scores with no differences in caffeine-treated versus placebo groups.^22^

**Fig. 1:**
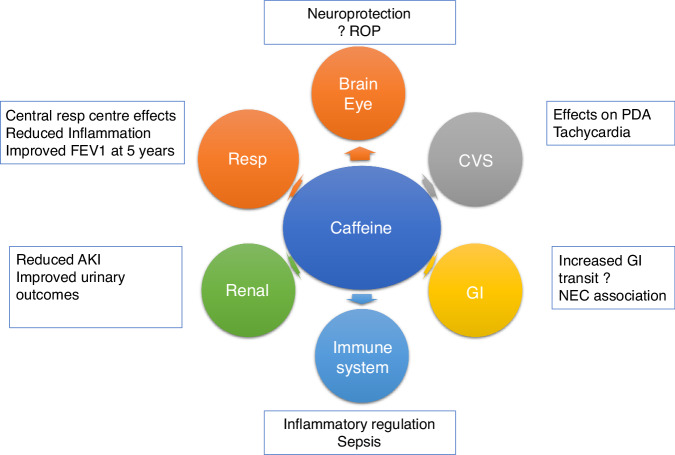
The effects of caffeine on multiple organ systems in neonates. The circles indicate the organ systems that caffeine affects, and the boxes beside each circle indicate these specific beneficial effects. PDA patent ductus arteriosus, ROP retinopathy of prematurity, NEC necrotising enterocolitis, AKI acute kidney injury.

Post hoc analysis of the CAP trial revealed a significant reduction in the incidence of neurodevelopment disability at 18–20 months of age^23,24^. The incidence of cerebral palsy at this point was 4.4% in the caffeine arm and 7.3% in the placebo (*p* value: 0.009%), and the incidence of cognitive delay was 33.8% versus 38.3%. However, by 5 years of age caffeine therapy was no longer associated with a significant reduction in neurodevelopmental disability. Caffeine therapy for AOP did not significantly reduce the combined rate of academic, motor, and behavioural impairments but was associated with a reduced risk of motor impairment in 11-year-old children with very low birth weight (VLBW).^19^

Mike et al. found that, in neonatal lambs exposed to HI, prenatal and postnatal caffeine administration was associated with significant reductions in proinflammatory cytokines, neuroinflammation, and grey matter injury with improved neurodevelopmental outcomes. Caffeine was well tolerated but showed toxicity at high doses.^25^ Early caffeine citrate use in animal models of neonatal HI is neuroprotective and mechanisms include adenosine receptor antagonism, phosphodiesterase inhibition, calcium ion activation and γ-aminobutyric acid receptor antagonism.^26–28^ Di Martino et al. found decreased proinflammatory cytokines, decreased brain injury markers on post-mortem samples, and improved behavioural assessment 2 weeks after HI in a neonatal animal model.^26^ This injury was normalised if caffeine was administered immediately after the injury but had no effect with later administration compared to the vehicle group. Endesfelder et al. found that caffeine reduced oxidative stress and inflammation in rat pups following hyperoxia and decreased pro-apoptotic factors and diminished extracellular matrix degeneration, and inhibitor of metalloproteinase.^27^ Therefore, the animal studies demonstrate the inflammatory response associated with caffeine in more detail as a mechanism for pulmonary and systemic inflammation.

A recent systematic review and meta-analysis of caffeine use and neurodevelopmental outcome demonstrated that although caffeine decreased apnoea and bronchopulmonary dysplasia (BPD) especially with higher doses there was no effect on early childhood neurocognitive impairment with moderate certainty of benefit in middle childhood motor function.^29^

### Caffeine for AOP

Caffeine citrate is the most commonly used methylxanthine for AOP as it is cost-effective, with excellent bioavailability, a wide therapeutic range, longer half-life, and minimal side effects. The cost-effectiveness analysis from the CAP study showed caffeine to be a dominant or “win–win” therapy with better outcomes and lower mean costs, although subsequent research has shown an increase in the cost of caffeine.^30,31^ In lower- and middle-income countries, high prices for caffeine and poor availability are commonly described barriers even though caffeine is on the essential drug list of the World Health Organisation (WHO).^32^

Study comparisons are complicated by changes in terminology. Caffeine citrate is the current form of caffeine used in neonatology—2 mg of caffeine citrate being equivalent to 1 mg of caffeine. A 2001 Cochrane neonatal review concluded that methylxanthines “reduced apnoea frequency and need for mechanical ventilation during the first seven days of therapy”.^4,5^ Subsequently, the CAP trial enrolled 2006 preterm infants weighing between 500 and 1250 g to evaluate short- and long-term benefits or risks of caffeine therapy beyond the first 7 days of treatment. Patients were enrolled if their clinicians considered them candidates for therapy. Clinician’s reasons included to prevent apnoea, to treat apnoea, and to facilitate extubation.^2,33^ Caffeine reduced the rate of BPD and retinopathy of prematurity in these patients but had no significant effect on mortality, brain injury or necrotising enterocolitis (NEC).^2,5^ Caffeine citrate is administered conventionally as a loading dose usually of 20 mg/kg followed by a maintenance daily dose of 5 mg/kg.^33^

RCTs have shown that caffeine reduced rates of extubation failure.^5^ Prophylactic caffeine therapy was associated with a significant reduction in extubation failure within the first week, likely as a result of an increase in central respiratory drive,^5,29,34^ Amaro et al. cast doubt on the efficacy and safety of early caffeine and ventilation weaning in preterm infants <30 weeks. This randomised, double-blind, placebo-controlled trial aimed to evaluate the efficacy of early caffeine on the age of first successful extubation in preterm infants^35^ that required mechanical ventilation within the first 5 days of life, who were randomised to receive early bolus and maintenance caffeine therapy or placebo until extubation.^35^ Initiation of early caffeine did not reduce the age of first successful extubation, rates of BPD, or the duration of need for supplemental oxygen when compared with the placebo group.^35^ Importantly, the trial was stopped early due to a non-significant trend towards an increased mortality rate in the caffeine group.^35^ The investigators did not report on neurodevelopmental outcome data post intervention.

Caffeine also reduced the rate of postoperative apnoea and bradycardia in preterm infants (born at 30–32 weeks gestation) when given either before or during anaesthesia induction at doses between 5 and 10 mg/kg.^5,36^ In a meta-analysis of three trials by Henderson-Smart et al., caffeine use was associated with a significant reduction of postoperative apnoea and bradycardia in preterm infants who had previously experienced apnoea. It was suggested that caffeine should be used to prevent postoperative apnoea, particularly in high-risk groups with previously unexplained apnoea, BPD and younger gestational age. However, the evidence base does not show efficacy in preterm neonates without risk factors.^5,36^

### Caffeine, pulmonary inflammation and respiratory outcomes

The beneficial effects of caffeine on neonatal respiratory function include multiple mechanisms, including anti-inflammatory properties, enhanced diaphragmatic function and activation of medullary respiratory centres. BPD has been associated with pulmonary and systemic inflammation, which may be exacerbated by episodes of hypoxia and hyperoxia associated with AOP. Nagatomo et al. demonstrated that in preterm rabbits, caffeine reduced the functional, architectural and inflammatory pulmonary changes induced by hyperoxia in the lung.^37^ Caffeine and hyperoxia upregulated hypoxia inducible factor-2α and vascular endothelial growth factor gene expression. Caffeine improved vascular remodelling decreasing pulmonary smooth muscle arteriole thickness following hyperoxia especially in male mice.^38^ In addition to results from the CAP trial, various caffeine dosing trials suggest a dose–response relationship with extubation success and possibly lung disease. Cochrane has reviewed dosing trials and suggested there may be a reduction in BPD from high-dose regimens (moderate-certainty evidence).^39^

The CAP trial compared caffeine citrate, at a loading dose of 20 mg/kg/day and maintenance dose of 5–10 mg/kg/day, to placebo in the treatment of AOP. There was a significant reduction in the rate of BPD and in the duration of positive pressure ventilation at 1 week.^2^

In a subgroup of children from the CAP trial (*n* = 142), expiratory flow measurements were better in the caffeine-treated group compared to placebo and fewer had forced vital capacity less than the 5th centile at the age of 11 years.^40^ Therefore, caffeine appears protective in hyperoxia resulting in improved pulmonary outcomes extending to middle childhood.

Bruschettini et al. in their recent Cochrane study reviewed dosing trials and suggested there may be a reduction in BPD from high-dose regimens (moderate-certainty evidence) but cautioned about caffeine use in the first hours of life when the risk for intracranial haemorrhage is the greatest. In addition, there is a paucity of data on extremely preterm infants, which is an important focus for future studies as well as longer time neurodevelopmental outcome data from completed trials.^39^ However, in the Cochrane study also in 2023 from Marques et al. there were 18 studies (2705 infants) included to assess the use of methylxanthine in preterm infants for: any indication (one study); prevention of apnoea (six studies); treatment of apnoea (five studies); and to prevent re-intubation (six studies). This study found that caffeine reduced death, major neurodevelopmental disability, reduced risk of any apnoeic episodes, cerebral palsy, developmental delay, and need for positive-pressure ventilation once initiated and chronic lung disease.^41^

### Use in weaning mechanical ventilation

Amaro et al. in an RCT showed no change in age at first extubation in the caffeine arm (median, 24 days; interquartile range (IQR), 10–41 days *n* = 41) compared to the placebo arm (median, 20 days; IQR, 9–43 days; *p* =  0.7; *n* = 42). An interim analysis performed at 75% enrolment showed a nonsignificant trend towards increased mortality in infants receiving caffeine prior to extubation.^36^ Another RCT compared adding an additional maintenance dose of caffeine citrate injection at 1 h before ventilator weaning. It was concluded that it was safe and effective in improving the success rate of ventilator weaning in preterm infants with respiratory distress syndrome.^42^

### Caffeine and renal function

In the Assessment of Worldwide Acute Kidney Injury Epidemiology in Neonates (AWAKEN) group (*n* = 675), administration of caffeine was associated with reduced odds of developing acute kidney injury (AKI; adjusted odds ratio, 0.20; 95% confidence interval, 0.11–0.34). The authors found that one case of AKI was prevented for every 4.3 neonates treated with caffeine. In addition, infants with AKI who received caffeine had a reduced risk of progressing to Stage 2 or 3 AKI. This corroborates previous retrospective work showing that in 140 VLBW infants caffeine exposure reduced the risk of AKI.^43^ In addition, caffeine use in preterm infants with NEC or spontaneous intestinal perforation (SIP) was associated with reduced incidence and severity of AKI.^44^ Increased renal tissue oxygenation in preterm neonates was found following caffeine in those low baseline values under 40% especially in the first 3 h after intravenous (IV) caffeine. Caffeine has been associated with increased urinary output in ventilated preterm lambs.^45,46^ Therefore, caffeine may have beneficial effects and have a role in the prevention of AKI in preterm infants but requires further study for correct timing and dosage.^47^

### Caffeine and gastrointestinal effects including NEC

Preterm infants randomised to early caffeine administration (<24 h) had decreased mesenteric tissue oxygenation compared to later administration and may be associated with the development of NEC.^48^ However, the CAP trials and other RCTs have not demonstrated an increase in SIP or NEC.^2^ Caffeine administration has been associated with an initial reduction in weight gain, which was greatest at 2 weeks, with a mean weight loss of 23 g.^5^ However, at 18–21 months no difference in weight gain was observed between trial arms.^5^ Although studies have postulated a reduction in intestinal and cerebral blood flow, no significant risk of NEC has been demonstrated.

### Caffeine, cardiovascular function and patent ductus arteriosus (PDA)

Caffeine increases the release of catecholamines in the bloodstream altering cardiac autonomic modulation and inducing consequent tachycardias. Caffeine and heart rate (HR) variability were studied in a systematic review showing increases in vagal flow via frequency domain indexes, but that result does not allow for exercise interventions. In neonates, Huvanandana et al. showed that caffeine not only affects and increases the HR and beat-to-beat pulse pressure variability but also has a definite effect on blood pressure although the exact mechanism is unclear. Caffeine is a global adenosine receptor antagonist and therefore has a role in regulating the immune response to sepsis and HI.^25^ Caffeine decreases endotoxin-induced release of cytokines and improves endotoxin-induced increases in plasma catecholamines.^49–51^

Caffeine inhibits prostaglandin production and activation and also has a diuretic effect. This may be the mechanism whereby there was a decreased incidence of PDA and need for medical or surgical management in the caffeine-treated infants in the CAP trial. Only 4.5% of the infants in the caffeine group required PDA ligation, which was significantly less than the placebo group (12.6%; *p* < 0.001). In preterm infants <28 weeks gestation (*n* = 423), 7–13 days of exposure to a moderate–large PDA significantly increased the incidence of BPD/death. However, in the PDA-TOLERATE trial, a persistent moderate-to-large PDA was not associated with an increased risk of BPD unless the infant required ≥10 days of intubation. In this group, prolonged PDA exposure (≥11 days) was associated with an increased risk of moderate/severe BPD. Preterm infants treated with early caffeine <3 days had increased PDA closure and decreased IVH. The exact mechanisms are unknown as in vitro there was no direct effect on ductal closure, but it may be related to inhibition of prostaglandins and immunomodulatory effects.

#### Caffeine, inflammation and sepsis

Caffeine also modulates the release and metabolism of other central neurotransmitters, including dopamine, acetylcholine, noradrenaline and serotonin.^5–7^ It has also been linked with anti-inflammatory effects at therapeutic doses, with treated infants displaying decreases in interleukin (IL)-6 and tumour necrosis factor alpha (TNF-a) and increases in IL-10 levels.^5,7^ However, caffeine has been shown to induce inflammation when used outside the therapeutic range.^5^

Decreased IL-10 has been found in tracheal aspirates in preterm babies with evolving BPD denoting a decrease in anti-inflammatory cytokines.^52^ Caffeine is known to be immunomodulatory and decreases lymphocyte cytokine production as well as modulates TNF-a production from cord blood monocytes.^53^ In preterm infants, IL-10 decreased in the first 24 h following the caffeine loading dose in both serum and tracheal aspirates. TNF-a, IL-1b and IL-6 increased after 1 week of caffeine treatment indicating a pro-inflammatory profile.^53,54^ In prenatally endotoxin-exposed rats with amnionitis, caffeine decreased pulmonary pro-inflammatory markers and associated improvement in lung function.^55^ Benefit was only found in rat pups with an inflammatory intrauterine environment indicating a potential improvement especially for preterm infants with chorioamnionitis. More research on the immunomodulatory effects of caffeine and its analogues is indicated as this may alter dosing and timing of administration.

In the diagnosis of neonatal sepsis using HR variability analysis caffeine altered the results reducing the benefits of this technique.^56^ In an animal model of sepsis although caffeine increased the HR it did not reduce mortality in sepsis.^57^ The anti-inflammatory effects of caffeine may have immunomodulatory potential in the management of neonatal sepsis. A synthetic methylxanthine pentoxifylline has demonstrated promise in the treatment of NEC and sepsis and is under study in several multination clinical trials.^58^

## Efficacy

### High-dose versus standard-dose caffeine

A systematic review and meta-analysis compared the use of high-dose caffeine versus standard-dose caffeine therapy in premature infants <32 weeks gestational age (6 RCTs *n* = 620).^59–65^ and found a reduced risk of BPD and mortality with high-dose caffeine regimen. However, no recommendations were made due to the considerable variance in the dosages and variance in duration of therapy used in the reported trials (Table 1). There may be cause for concern with higher doses of caffeine citrate (80 mg/kg).^54^ Higher doses of caffeine were hypothesised to further improve white matter function in an RCT but those neonates receiving a high loading dose of 80 mg/kg had a higher incidence of cerebellar injury with subsequent alterations in early motor performance.^64^ Adjusting maintenance doses is suggested, as metabolism of caffeine increases with advancing postnatal age.^66^ National guidelines all recommend standard dosages as used in the CAP trial.^56,58^

**Table 1:** Variance in dosage regimens in high-dose RCTs.

	Romangnoli, et al.^60^	Scanlon et al.^61^	Gray et al.^62^	Steer et al.^63^	Mohammed et al.^65^	McPhearson et al.^64^
High dose (mg/kg/day)	LD:10	LD:50	LD:80	LD:60	LD:40	LD:80
	MD:5	MD:12	MD:20	MD:30	MD:20	MD:10
Low dose (mg/kg/day)	LD:10	LD:25	LD:20	LD:6	LD:20	LD:30
	MD:2.5	MD:6	MD:5	MD:3	MD:10	MD:10

*LD* loading dose, *MD* maintenance dose.

In a recent RCT, Zhang et al. compared the use of low (5 mg/kg/day) caffeine citrate to a higher dose (10 mg/kg/day). The results show that higher maintenance doses are more effective, not only shortening the duration of apnoea and reducing the use of invasive respiratory support, but also have no significant adverse effects on the treatment outcome of VLBW preterm infants.^67^

At standard doses such as those used in the CAP trial, caffeine is well tolerated.^5^ At high loading doses (e.g., 80 mg/kg IV) there is need for caution. A recent RCT (*n* = 74) randomly assigned either a high (80 mg/kg IV) or standard (20 mg/kg IV) dose of caffeine citrate to preterm infants <32 weeks gestation who presented with AOP within the first 10 days of life. Follow-up revealed a significant increase in the incidence of cerebellar haemorrhage on MRI (36% versus 10%, *p* = 0.03) and increased hypertonia summary scores on the NICU Network Neurobehavioral Scale (2.3 versus 1.5, *p* = 0.02) in the high dose group, so that the authors recommended against any further trials using such dosage.^65^

Metabolic acidosis and hyperglycaemia have been reported in acute caffeine toxicity and accidental overdose.^68^ Most published data do not endorse routine therapeutic drug monitoring. When caffeine is given at standard dose, serum levels remain within therapeutic range (5.5–23.7 mg/L) but if higher doses are needed, this will need to be reassessed.^69^ There were no trials of high-dose versus low-dose caffeine that reported on neurocognitive impairment.^29^

Therapeutic creep is described in the use of high-dose caffeine. Considerable variation in practice on caffeine use was noted across the UK and included 92 neonatal units.^70^ Although all units used caffeine less than half initiated it within 24 h of birth and discontinued at 34 weeks gestational age irrespective of respiratory status. This survey highlighted major differences in initiation of caffeine, gestation at routine administration and discontinuation.

### Prophylactic caffeine

A 2010 Cochrane neonatal review concluded that there was insufficient evidence to support the use of prophylactic methylxanthines in preterm infants who were considered high risk for developing apnoea.^71^ The review included three studies,^5,72,73^ comparing patient groups that had been randomly or quasi randomly allocated prophylactic caffeine to those who received placebo or no treatment at all. There was no significant difference in apnoea, bradycardia, hypoxemic episodes, or the use of IPPV.^71^ Prophylactic high dose methylxanthine therapy is not supported by the evidence and the effects of methylxanthine prophylaxis require further examination in high-risk preterm neonates.^71^ Caffeine continues to be used prophylactically in prematurity despite the lack of evidence.^74^

In several instances, caffeine used prophylactically improved respiratory effort when corrected for gestational age in animal and human trials.^8,75^ This effect is demonstrated via an improved tidal volume,^75^ increased diaphragmatic activity and tidal volume.^8^ A recent RCT demonstrated an increase in respiratory effort through these metrics when caffeine was given directly after birth. However, it did not show any change in clinical outcomes. These authors recommended that a larger study size was needed to find effects.^76^

Caffeine continues to be used as prophylaxis. Indications for prophylaxis vary but are commonly based on birth-weight or gestational age. A 2011 survey of 52 neonatal units in England found that 47% of units started caffeine therapy based on gestational age, irrespective of respiratory status.^68^ With the exception of the CAP trial, which compared caffeine to placebo, there is a lack of evidence comparing prophylactic caffeine to therapeutic caffeine in neonates.^24^

### When to stop caffeine?

Common clinical practice is to stop caffeine therapy when episodes of Apnoea, bradycardia and desaturations are resolving, typically around 34 weeks postmenstrual age (PMA). A recent RCT showed no difference in the recurrence of AOP when caffeine was stopped at 7-day apnoea-free period versus at 34 weeks PMA. Larger trials are required that specifically study extremely preterm infants in order to make robust recommendations on when to stop therapy.^77^ Substantial intermittent hypoxia (IH) persists after discontinuation of routine caffeine treatment and progressively decreases with increasing PMA. Extended caffeine treatment beyond 35 weeks of gestation decreases IH in premature infants <32 weeks in an RCT.^78^ A Cochrane review of cessation of caffeine including 3 RCTs with (*n* = 392 infants) concluded that all-cause mortality and apnoea had little or no difference in infants who were randomised to later discontinuation of caffeine treatment.^79^ Later cessation resulted in a reduced number of infants with at least one episode of IH. However, there was no research on adverse or positive effects of later caffeine discontinuation, regarding recommencing caffeine or need for respiratory support(invasive or non-invasive) within a week of discontinuation.

### Guidelines

Guidelines from the Royal Children’s Hospital Melbourne,^74^ the NHS South West Neonatal Network,^80^ the South Australian Neonatal Medications Group,^77^ the Auckland District Health Board,^81^ Northern California Neonatology Consortium,^82^ World Health Organisation Pocketbook of Hospital Care for Children,^83^ European Consensus Guidelines on the Management of Respiratory Distress Syndrome^84^ (Table 2) and the National Institute for Health and Care Excellence recommend a maintenance dose of 5–20 mg/kg/day of caffeine citrate but higher doses can be used if therapeutic efficiency is not achieved while ensuring a safe plasma level is maintained.^85^ All recommend the same loading and maintenance dose of 20 and 5 mg/kg of caffeine citrate, respectively, but there is variation in indications for prophylaxis and criteria for discontinuation. The wide variation in international guidelines has been highlighted in several articles and consensus on dose and time of initiation of therapy is needed.^86^

**Table 2:** Summary of guidelines on caffeine.

Guidelines	Loading dose	Maintenance dose	Indications for prophylaxis	Criteria for discontinuation
WHO^83^	20 mg/kg	5–20 mg/kg	Yes, dates not specified	Not specified
European Consensus^84^	20 mg/kg	5–10 mg/kg	Yes, dates not specified	Not specified
NICE^85^	20 mg/kg	5–20 mg/kg^a^	<30 weeks,	33−35 weeks cGA
AAP	20 mg/kg	5–10 mg/kg	Not stated	No apnoea/bradycardia off positive pressure for 5−7 days, OR at 33−34 weeks
RCH Melbourne^74^	20 mg/kg	5 mg/kg	<34 weeks	34 weeks
NHS SWNN^80^	20 mg/kg	5–10 mg/kg	<30 weeks OR <1.5 mg OR on CPAP	No airway support OR apnoea >5 days
SANMG^81^	20 mg/kg	5–10 mg/kg	Not stated	Not stated
ADHB^87^	20 mg/kg	Not stated	Subjective clinical assessment	32–34 weeks AND 5 days without apnoea
UCSF NCNC^82^	20 mg/kg	5 mg/kg	<30 weeks	<26 weeks: 36 weeks OR >35 weeks apnoea free> 3 days <2 weeks from discharge >26 weeks: 33–34 weeks if 3 days apnoea free and <2 weeks from discharge

*WHO* World Health Organisation, *NICE* National Institute for Health and Care Excellence, *AAP* American Academy of Paediatrics, *RCH* Royal Children’s Hospital, *NHS* *SWNN* National Health Service South West Neonatal Network, *SANMG* South Australian Neonatal Medication Guidelines, *ADHB* Auckland District Health Board, *UCSF NCNC* Northern California Neonatology Consortium, *cGA* corrected gestational age.

^a^Consider maintenance doses higher than 20 mg/kg daily if therapeutic efficacy is not achieved while ensuring that a safe plasma level is maintained.

## Conclusions

At current recommended doses, caffeine is a safe and effective therapy for the treatment of AOP. It has also been shown to have multiorgan effects with potential renal and neuroprotective benefits. However, the evidence is limited by a relative deficiency of well-powered RCTs and heterogeneity in reported outcomes within the current body of evidence. Trials to determine the optimal dose of caffeine should proceed with caution, given the potential for harm highlighted in the literature. All guidelines recommended prophylaxis despite the lack of a definitive evidence base. In view of the widespread use of caffeine, international consensus guidelines would be ideal.
